# Molecular Mechanisms of Biofilm Formation in *Helicobacter pylori*

**DOI:** 10.3390/antibiotics13100976

**Published:** 2024-10-16

**Authors:** Kartika Afrida Fauzia, Wiwin Is Effendi, Ricky Indra Alfaray, Hoda M. Malaty, Yoshio Yamaoka, Muhammad Mifthussurur

**Affiliations:** 1 Research Center for Preclinical and Clinical Medicine, National Research and Innovation Agency, Bogor 16915, Indonesia; kartikafauzia@gmail.com; 2 *Helicobacter pylori* and Microbiota Study Group, Institute of Tropical Disease, Universitas Airlangga, Surabaya 60131, Indonesia; 3 Department of Pulmonology and Respiratory Medicine, Faculty of Medicine, Universitas Airlangga, Surabaya 60131, Indonesia; 4 Department of Environmental and Preventive Medicine—The Research Center for GLOBAL and LOCAL Infectious Disease (RCGLID), Faculty of Medicine, Oita University, Yufu 879-5593, Japan; rickyindraalfaray@gmail.com (R.I.A.); yyamaoka@oita-u.ac.jp (Y.Y.); 5 Department of Medicine, Baylor College of Medicine, Houston, TX 77030, USA; hmalaty@bcm.edu; 6 Division of Gastroentero-Hepatology, Department of Internal Medicine, Faculty of Medicine—Dr. Soetomo Teaching Hospital, Univcersitas Airlangga, Surabaya 60286, Indonesia

**Keywords:** biofilm formation, *H. pylori*, infection, treatment, human and health

## Abstract

Background: Biofilm formation in *Helicobacter pylori* (*H. pylori*) helps bacteria survive antibiotic exposure and supports bacterial colonization and persistence in the stomach. Most of the published articles have focused on one aspect of the biofilm. Therefore, we conducted the current study to better understand the mechanism of biofilm formation, how the biofilm contributes to antibiotic resistance, and how the biofilm modifies the medication delivery mechanism. Methods: We conducted a literature review analysis of the published articles on the *Helicobacter pylori* biofilm between 1998 and 2024 from the PubMed database to retrieve eligible articles. After applying the inclusion and exclusion criteria, two hundred and seventy-three articles were eligible for our study. Results: The results showed that biofilm formation starts as adhesion and progresses through micro-colonies, maturation, and dispersion in a planktonic form. Moreover, specific genes modulate each phase of biofilm formation. Few studies have shown that mechanisms, such as quorum sensing and diffusible signal factors, enhance coordination among bacteria when switching from biofilm to planktonic states. Different protein expressions were also observed between planktonic and biofilm strains, and the biofilm architecture was supported by exopolysaccharides, extracellular DNA, and outer membrane vesicles. Conclusions: This infrastructure is responsible for the increased survival of bacteria, especially in harsh environments or in the presence of antibiotics. Therefore, understanding the biofilm formation for *H. pylori* is crucial. This study illustrates biofilm formation in *H. pylori* to help improve the treatment of *H. pylori* infection.

## 1. Introduction

**Figure 1:**
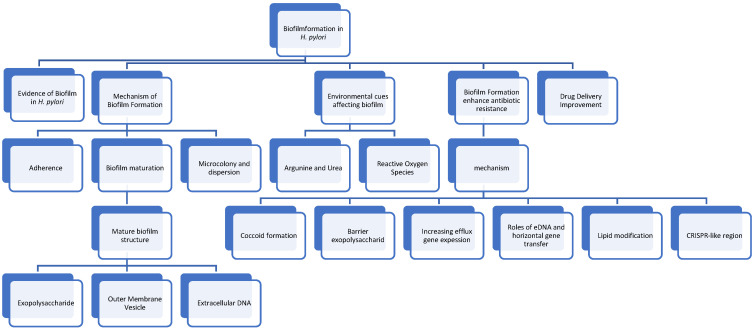
A Conceptual framework of *Helicobacter pylori* biofilm formation mechanism and eradication strategies. The central theme is the interplay between various factors influencing biofilm development and the need for innovative approaches to disrupt biofilm structure and enhance antibiotic susceptibility.

**Figure 2:**
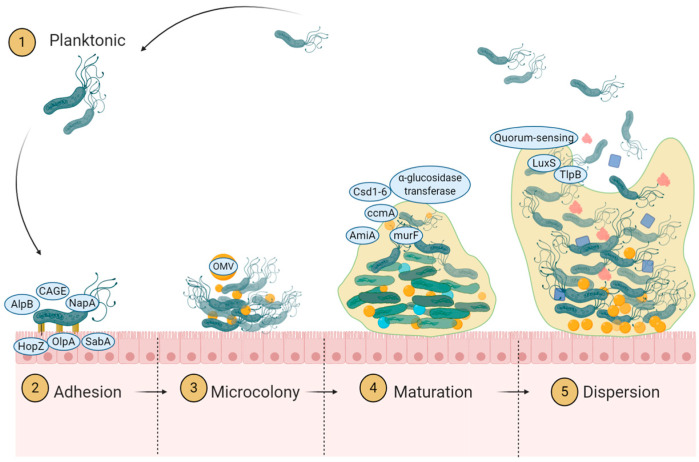
Graphic summary of biofilm formation mechanism.

The current state of the research field should be *H. pylori* is well-known as a pathogenic bacterium that causes chronic gastro-duodenal diseases, such as gastric ulcer, duodenal ulcer, and gastric cancer [1]. The strong relationship of *H. pylori* with gastric cancer, evidenced by numerous cohort and prevalence studies, led the International Agency for Research on Cancer to categorize *H. pylori* as a class I carcinogen. A high rate of infection increases gastric cancer morbidity and mortality [2,3]. The eradication of *H. pylori* is essential to reduce gastric ulcer, duodenal ulcer, and gastric cancer incidence [4]. However, emerging antibiotic resistance in several countries even after following treatment guidelines introduces obstacles for eradicating the infection [5]. Mechanisms for antibiotic resistance to *H. pylori* indicated a multiple contributing factors. Studies reported including reduced permeability, increased efflux pumping, mutation of target genes, direct modification of antimicrobial agents, and biofilm formation [6,7]. Biofilm is also an unpredictable factor that can substantially increase the concentration of mutations and resistance. Each year, new studies reveal findings that enhance the understanding of the mechanisms underlying biofilm formation. Currently, the standard triple therapy using clarithromycin and amoxicillin are still the first regiment used [8]. An update on the comprehensive summary of those factors has not been conducted. Therefore, we conducted this extensive review to update our understanding of *H. pylori* biofilm formation to help in finding targets for biofilm eradication and enhance therapeutic efficacy. (the outline is depicted in Figure 1 and Figure 2 explained the biofilm formation cycle).

## 2. Search Method

We conducted a literature review analysis of the published articles on *Helicobacter pylori* biofilm between 1998 and 2024 from the PubMed database to retrieve eligible articles. We searched the PubMed database using the keywords (“*Helicobacter pylori*”) AND (Biofilm) up to June 2024 and obtained 273 articles. From the 273 articles, we narrowed down the search using keywords (“*Helicobacter pylori*”) AND (biofilm)) AND (antibiotic)) AND (resistance) AND ((“Helicobacter pylori”) AND (biofilm)) AND (mechanism), obtaining 108 articles and 71 articles, respectively. The exclusion criteria are articles that do not explain biofilms and biofilms from other species (Table S1).

## 3. Evidence on How *H. pylori* Forms Biofilm

*The H. pylori* organism exists in planktonic (solitary) and biofilm aggregates, which shows the adaptation of *H. pylori* in high-stress environments. *H. pylori* is a motile bacterium with multiple flagella [9] that matches the organism’s planktonic form. However, an unfavorable environment triggers biofilm formation and an aggregate or colony of sessile bacteria. The main features of biofilms are heterogeneity, slow growth, stability, and resistance to eradication [10]. Biofilm production allows for many strains within a single species, numerous species, and even multiple kingdoms within a single structure. Organisms reside within the adhesive structure of exopolysaccharides (EPSs) and, frequently, extracellular DNA (eDNA). This form provides a best-fit place for bacterial survival in stressful environments, including high acidity and the presence of antimicrobial agents. This profile may extend some environments’ colonization periods [11].

*H. pylori* biofilm development was observed in vivo using human stomach biopsy specimens and imaged using scanning electron microscopy. The study revealed that individuals with peptic ulcer disease who tested positive for urease had a biofilm coverage of 97.3%, whereas *H. pylori*-negative patients had a biofilm coverage of 1.64% [12,13]. The visualization of biofilm formation in gastric samples could also be evaluated via immunohistochemistry [14].

Mouse stomachs also clearly exhibit the formation of biofilm [15]. In an ecological context, it has been reported that *H. pylori* may be present in water distribution networks [16,17]. Moreover, it can be found in biofilms that form on the inner surfaces of pipework in drinking water systems, and it can attach itself to various types of materials [18,19,20].

Meanwhile, in vitro studies reported that biofilms grown on cover glass can demonstrate biofilm formation in 72% of the strains evaluated, while another study showed a biofilm in 93.1% [21].

The biofilm’s morphology and cell interaction can be visible on an electron micrograph. In SEM images, at the initial stage of the biofilm, *H. pylori* is rod-shaped but rarely has flagella. At a further stage, the coccoid-shaped bacteria pile up and are covered with polysaccharides [22]. In addition to scanning electron microscopy (SEM), biofilms can be observed using confocal laser scanning microscopy (CLSM). Using CLSM makes it possible to visualize biofilms on biotic surfaces, such as AGS cells. Biofilms can be stained with fluorescence, and a biofilm can be seen on the entire surface of the AGS cells [23]. Biofilms are, thus, important for the survival of *H. pylori* in many environments. Several steps or phases were reported to form a complex biofilm because it is not as simple as toxin excretion but requires the interaction of a single bacterium with the surface and other bacteria [24]. 

## 4. Mechanism of Biofilm Formation

### 4.1. Initiation of Adherence

#### 4.1.1. Role of Adhesion Protein

The biofilm formation process relies heavily on adherence, necessitating a stable foundation surface. In particular, an adhesion protein is key in attaching to the gastric epithelial surfaces and promoting biofilm formation. The *alpB* gene is responsible for initiating the adherence of *H. pylori*, and when this gene is knocked out, there is a reduction in biofilm formation [25]. It is also reported that *cagE* is involved in biofilm formation [26], and it is located on cag pathogenicity islands, supporting cagA translocation to gastric cells [27]. *cagE H. pylori* adhesion to the AGS cell is less than the wild type, leading to lower biofilm formation [28]. Strains lacking other adhesion proteins, such as OipA and SabA, produce less biofilm.

#### 4.1.2. Role of Mucin Binding Protein 

The human gastrointestinal tissue is coated with several mucins. NapA is a protein that specifically binds to sulfated oligosaccharides in *H. pylori*, and its primary function is to attach to mucin [29,30]. A transcriptomic study reported that NapA expression was upregulated in biofilm cells [31], and another study reported that strains lacking HopZ produce more biofilm [32].

#### 4.1.3. Role of Chemotaxis

During the adherence process, chemotaxis is the first stage of biofilm formation but does not affect the production of mature biofilms. A study examining various chemotaxis signaling mutants found that chemotaxis proteins are involved in biofilm initiation by affecting flagellar rotation [33]. Moreover, mutants that preferred counterclockwise movement encouraged the formation of biofilms, such as ∆*cheA*, ∆*cheW*, or ∆*cheV*. On the other hand, mutants that preferred clockwise movement hindered the formation of biofilms, such as ∆*cheZ*, ∆*chePep*, or ∆*cheV3*. These counterclockwise (CCW) flagella were inadequate in stimulating the production of biofilms, indicating the presence of other factors responsible for this process [33].

### 4.2. Microcolony Formation and Dispersion 

#### 4.2.1. Definition of Microcolony Formation

One key aspect of biofilm formation is the early development of microcolonies, where approximately 100 cells cluster together without clear borders, overlapping with the initial attachment phase [10]. These microcolonies serve as the foundation for biofilm formation, which requires bacteria to group, either as a single or multiple species, to create a community that offers protection, nutrients, and optimal conditions for survival. Bacteria within biofilms sense their environment and adjust metabolic processes to utilize available resources efficiently. The limited oxygen diffusion within biofilms creates distinct microenvironments that accommodate aerobic, facultative anaerobic, and obligate anaerobic bacteria [34]. Within biofilms, bacteria exhibit cooperative and altruistic behaviors facilitated by a process known as quorum sensing [35]. 

#### 4.2.2. Role of Quorum Sensing

Quorum sensing is a molecular mechanism that enables cell-to-cell communication by producing signaling molecules called autoinducers. At least two substances are crucial in quorum sensing and signaling communication: autoinducer-2 (AI-2) and diffusible signal factor. These molecules regulate gene expression upon reaching a certain threshold, influencing bacterial adherence, aggregation, spatial organization, and even dispersal from the biofilm [35]. LuxS, a key enzyme in the activated methyl cycle, produces the quorum sensing molecule autoinducer-2 (AI-2) in *H. pylori* [36,37]. During microcolony formation in *H. pylori*, LuxS-mediated AI-2 signaling plays a pivotal role in coordinating bacterial communication and regulating gene expression in response to cell density [35,38,39]. These reports indicate that the production of AI-2 by *luxS* is growth phase-dependent, with maximal production occurring in the mid-exponential phase of growth. AI-2 is also involved in the structure and function of flagella. The mutant of *luxS* affects the flagella synthesis by *the flaA*, *flgE*, *motA*, *motB*, *flhA*, and *fly* genes [40]. An in-vivo study showed the involvement of *luxS* in motility, ∆*luxS H. pylori* showed less ability to colonize [41]. Further relation of autoinducer and motility also showed by the study demonstrating *fur* (ferric uptake regulator) gene that affects the flagellar motor switch and the synthesis of autoinducer-2 (AI-2) [37].

A diffusible signal factor (DSF) is proposed as a communication molecule in biofilm formation. DSFs are a class of fatty acid derivatives implicated in inter- and intraspecies communication in the quorum sensing system [42]. This communication affects the production of extracellular enzymes. Yamashita et al. showed that cis-7-tetradecenic acid and lauric acid are DSF molecules in *H. pylori*. These DSFs are involved in auto-inhibitory substances, and they produce self-inhibiting substances with bacteriostatic activity and trigger *H. pylori* to change from a spiral into a coccoid form [43,44].

Dispersal is important for the distribution of sources, and a gradient might exist to strat-ify protection levels after the biofilm is no longer beneficial for the H. pylori survival. Periplasmic binding proteins AibA and AibB are required for AI-2 chemorepulsion re-sponses [35]. Autoinducer signaling also involves TlpB, a family of chemoreceptors. TlpB is a receptor of AI-2, and signaling is perceived as a chemorepellent. This activity could cause cells to disperse to find vacant space and nutrients [40]. The attachment of TlpB to the receptor would generate dispersal and determine the spatial organization of biofilm formation [35].

### 4.3. Biofilm Maturation

Biofilm maturation in *H. pylori* involves a significant morphological transformation accompanied by an increase in extracellular polymeric substance (EPS) synthesis [45]. This maturation process is closely linked to the formation of the extracellular matrix, which plays a crucial role in biofilm development [46]. Studies have shown that there is a notable difference in biofilm biomass production between 24 and 72 h, indicating a time-dependent maturation process [47]. Mutants with impaired motility or flagella production exhibit reduced biofilm formation capabilities, partly due to flagellar filaments within the biofilm structure [7].

*H. pylori* mutants with impaired motility (∆motB) or flagella production (∆*fliM*, ∆*fliA*) exhibit reduced ability to form mature biofilms, partly because of the presence of flagellar filaments in the biofilm structure [46]. Adjusting *H. pylori’s* phenotype during biofilm growth involves metabolic changes and the transformation of flagella into adhesive structures, further emphasizing the role of motility in biofilm development [48]. Biofilm formation in *H. pylori* has been associated with increased antibiotic resistance, with mature biofilms showing significantly higher resistance levels compared to planktonic cells [7]. This ability possessed by mature biofilm is contributed by the structure that consists of:

#### 4.3.1. Extracellular Polysaccharide Deposit

One component that supports EPS structure is mannose-related proteoglycan, termed proteomannans. Nuclear magnetic resonance analysis of biofilm EPS revealed 1-4 mannosyl linkages in developing and mature biofilms [31]. Monosaccharide analysis from the biofilm matrix purification discovered fucose (Fuc), galactose [36], glucose (Glc), glyceromanno-heptose [49], *N*-acetylglucosamine (GlcNAc) and N-acetylmuramic acid (MurNAc) as the main component. Amino acid and lipid, especially tetradenoic acid (C14) and hexadecanoic acid (C16) are also contributed to the biofilm architecture [50]. 

*N*-acetylglucosamine (GlcNAc) structure in the mature biofilm is also contributed to the microbiome environment. Study reported role *H. pylori* biofilm to the survival of Enter-ovirus 71 (EV71) in the environment. Results show that EV71 particles can persist for up to 10 days when incubated with *H. pylori* biofilm, and its viability is dependent on the quantity of biofilm formation. The biofilm contains N-acetyl-glucosamine and glycosaminoglycan, which has binding affinity to EV71 [51].

#### 4.3.2. Outer Membrane Vesicle as Extracellular Substance

Gram-negative bacteria, such as *H. pylori*, have been found to constitutively secrete outer membrane vesicles (OMVs) into the extracellular environment, which serve as es-sential mediators for intercellular communication, survival, and pathogenesis [52]. In the context of biofilm formation, a study on the TK1402 strain of *H. pylori* highlighted the significance of OMVs in this process [53]. These OMVs have been observed both on glass surfaces and bacterial surfaces, with transmission electron microscopy revealing their presence at the substratum–bacterium interface, suggesting a potential role as an extracellular polymeric substance (EPS) matrix for biofilm development [54]. The proteomic content of OMVs from Gram-negative bacteria has been a subject of interest, as these vesicles contain biologically active proteins that contribute to various biological processes [55]. Furthermore, OMVs have been associated with diverse functions, including promoting inflammatory responses in hosts, facilitating adhesion to host cells, and delivering virulence factors [56]. The ubiquity of OMV production among Gram-negative species underscores the importance of these vesicles in facilitating essential functions for organisms living in biofilm communities [57]. 

Moreover, OMVs have been implicated in cell-to-cell communication, genetic exchange, cargo delivery, and pathogenesis, highlighting their versatile roles in bacterial physiology and interactions [58]. The presence of OMVs within biofilm matrices, as demonstrated in various studies, suggests that these vesicles play a crucial role in biofilm structure and function [59]. Additionally, the association of OMVs with biofilm disper-sion and their impact on biofilm composition further emphasize the significance of these vesicles in the dynamics of bacterial biofilms [60]. The secretion of OMVs by Gram-negative bacteria, including *H. pylori*, represents a sophisticated mechanism for intercellular communication and biofilm formation. Understanding the roles and functions of OMVs in bacterial communities is essential for unraveling the complexities of biofilm development and bacterial pathogenicity. An alternative approach to decrease OMV release could involve the utilization of peptidyl arginine deiminase inhibitors [61]. This could make bacteria more susceptible to antibiotic treatment and potentially decrease the development of antibiotic resistance linked to the creation of biofilms. An alternative strategy could entail the utilization of quorum sensing inhibitors (QSIs) to control bacterial pathogenicity [62]. QSIs can disrupt biofilm development by targeting the signaling systems that are involved in the creation of OMVs. 

#### 4.3.3. Extracellular DNA

eDNA is a component of biofilm matrices and is important for stabilizing biofilm structure. This component is involved in other mechanisms, such as recombination, trans-formation, and genomic variability. Restriction Fragment Length Polymorphism (RFLP) analysis between intracellular DNA and extracellular DNA concludes that eDNA may not come from the lysis of cells but is purposively secreted to extracellular spaces [63]. eDNA is protected from DNase I and nuclease degradation by other extracellular polymeric substances [64]. Aggregation of OMV, as the early steps for biofilm formation, were also induced by eDNA [63]. The circulating DNA in the biofilm contributes to the higher recombination rate [65,66].

## 5. Environmental Cues Affecting Biofilm Formation

*H. pylori* are exposed to mechanical stress such as washing action, peristaltic movement and bulk food through the stomach. It is also prone to chemical stress such as low pH of gastric juice and nutrient deprivation [67]. In vitro study reported increasing biofilm formation in the exposure of *H. pylori* to sub-inhibitory concentration of antibiotics [66]. Dead cells, induction of stress responses, and physiological changes in injured cells might signal biofilm formation [68]. Yonezawa et al. explained exposure to minimum inhibitory concentrations of clarithromycin of *H. pylori* biofilm biomass by up to fourfold (2-day bio-film) and 16-fold (3-day biofilm). Another study also showed that sub-MIC doses of amoxicillin and clarithromycin exposure induce an increase in biofilm formation by *H. pylori* [69]. Beside the antibiotic’s exposure, several compounds in the environment could be the environmental cues for *H. pylori* for biofilm; including Arginine, Urea and Reactive oxygen Species are the most prominent. 

### 5.1. Arginine and Urea

Proteins involved in colonization, such as urease and ferritin, are upregulated in biofilm cells [31]. Urease and ferritin are important for acid environment conditioning before colonization. *H. pylori* utilize arginine and urea as key environmental cues in biofilm formation. The bacterium possesses multiple chemoreceptors, including TlpA, TlpB, TlpC, and TlpD, which are crucial in sensing these cues. Studies have shown that deleting specific chemoreceptors can impact biofilm formation differently. For instance, the strain lacking TlpA exhibited enhanced initial biofilm formation, while the strain lacking TlpB showed reduced biofilm formation [33].

It has been observed that arginine promotes the growth of biofilms, while urea hinders their development in *H. pylori*. This is significant as *H. pylori* possesses the rocF gene encoding arginase, which is involved in the pathogenesis of H. pylori infection [70]. Additionally, the urease enzyme in *H. pylori* catalyses the hydrolysis of urea to ammo-nia and carbon dioxide, which can influence biofilm development.

### 5.2. Reactive Oxygen Species

The host immune cells create reactive oxygen species (ROS) in response to *H. pylori* infection, but ROS cannot efficiently eliminate the bacterium. While ROS are produced as a defense mechanism against *H. pylori*, the bacterium can exploit the biofilm’s extracellular matrix to protect itself from the toxic effects of ROS. Studies have demonstrated that exposure to low levels of hydrogen peroxide (H_2_O_2_) can enhance biofilm production in *H. pylori*, indicating a complex interplay between ROS and biofilm formation [21].

Transcriptome analysis has identified genes such as *spoT* (HP0775) and *napA* associated with ROS response that play significant roles in biofilm enhancement. The *spotting* gene encodes a (p)ppGpp synthase/hydrolase enzyme, consisting of 26 amino acids; (p)ppGpp plays a role in governing the stringent/stress response in bacteria that are released in challenging conditions, including dietary shortage, thermal stress, and exposure to antibiotics. Additionally, the research indicates that SpoT-mediated upregulation of NapA promotes oxidative stress-induced biofilm formation in *H. pylori*. NapA, another gene implicated in the ROS response, has been shown to enhance biofilm production and confer resistance to various drugs in *H. pylori*. NapA protects against oxidative stress, shielding *H. pylori* from damage caused by ROS and contributing to biofilm development triggered by oxidative stress [71]. The Δ*napA* strain was found to have produced a thin biofilm [21]. The ΔnapA biofilm had a less compact bacterial structure, poor extracellular matrix production, greater cavities, and a spherical shape compared to the WT and napA* biofilms. The upregulation of NapA expression is the primary mechanism in *H. pylori* that counteracts the deficiency of significant oxidative stress factors. The *H. pylori napA* mutants have decreased survival rates when exposed to oxidative stress compared to the WT strain, which suggests that napA plays a protective role in defending *H. pylori* against damage caused by oxidative stress [71].

## 6. Biofilm Formation Enhances Antibiotic Resistance

Antibiotic resistance has emerged in various locations around the world. The most recent meta-analysis indicated a rising trend from 2013 to 2023 [72,73]. The highest prevalence was observed for metronidazole resistance, followed by clarithromycin, in the Southeast Asia, Europe, and Africa regions [72]. The prevalence of resistance to amoxicillin is low in most areas. However, it began to rise, particularly in DR Congo and Mongolia [74,75,76]. This outcome could be attributed to the presence of a biofilm. A prior investigation indicated that strains capable of biofilm formation exhibit a higher resistance level than those that do not form biofilms [75,77]. Antibiotic resistance is defined as increasing the minimum inhibitory concentrations of antibiotics due to a permanent change in cells, such as mutation or the acquisition of resistance functions by horizontal gene transfer [24,78].

A study reported high biofilm formers to increase bacterial survival during antibiotic exposure [77]. The minimum bactericidal concentrations of CLR against cells in the biofilm were higher (1.0 µg/mL) than for planktonic cells (0.25 µg/mL). *H. pylori* CLR resistance mutations are more frequently observed in biofilms than in planktonic cells [66]. The mechanisms of antibiotic resistance by biofilm formation are explained as follows:

### 6.1. Coccoid Formation

*H. pylori* can live in two forms: spiral actively dividing forms and nonculturable but viable coccoid forms. *H. pylori* cells can transform into coccoid forms within biofilms. The coccoid form is a viable but nonculturable state and less active metabolically, which can revive and cause the recurrence of the infection [79]. The coccoid forms reported in the study were detected using Gram staining and light microscopy, and their morphological modifications were validated [80], which can result in recurrent infections and relapses, even after attempts at eradication [81]. The term relapse is also called recrudescence, defined as the re-emergence of infection due to the survival of the original infecting strain. This differs from reinfection, where different strains infect an individual [80]. The existence of coccoid *H. pylori* within biofilms can potentially worsen chronic infections and impede the successful eradication of the bacteria. Coccoid variants of *H. pylori* develop in response to unfavorable environmental conditions, including food scarcity, antibiotic exposure, and pH and oxygen levels [82,83]. This activity is essential as it may result in varied levels of inflammation, which can sustain gastritis and induce relapses post-treatment [84,85].

It has been reported that exposure to metronidazole or clarithromycin in sub-MIC doses increases the change of cells into coccoid form. [86]. The coccoid type of bacteria possesses peptidoglycan modification and a mucoid covering that hinders the penetration of antibiotics. The molecular synthesis and metabolism are also reduced. The antibiotic works best for the bacteria in the lag and log phases [87]. Hence, the presence of “dormant” cells is a significant cause of the ineffectiveness of antibiotics against bacterial biofilms [88].

### 6.2. The Barrier of Extracellular Polysaccharide

Research has shown that exopolysaccharides play a crucial role in the biofilm matrix, providing structural stability to biofilms and shielding bacteria from antimicrobial substances [89,90]. Exopolysaccharides seen in biofilms have been associated with increased antibiotic resistance. They achieve this by obstructing the entry of antibiotics and forming a barrier that prevents phagocytosis by the host’s immune cells [49]. Exopolysaccharides play a dual role in biofilm formation by providing structural support and limiting the entry of antibiotics, therefore contributing to antibiotic resistance [91]. Gradually introducing antibiotics may allow for adaptive phenotypic responses, potentially enhancing tolerance [24,88,92].

### 6.3. The Increasing Expression of Efflux Gene

*The hefA* gene code for the efflux gene plays a vital role in multidrug resistance [93]. Attaran et al. investigated the efflux gene, *hp1165*, and *hefA* expression on the biofilm-forming cells compared to planktonic cells by quantitative PCR. The expression was significantly higher, and there was an association between biofilm formation and antibiotic resistance [94]. Increasing resistance–nodulation–division (RND) efflux pump gene expression on the biofilm forming was also reported [95]. Other efflux genes, *hp0939*, *hp0497*, and *hp0471*, were highly expressed in the clinical isolates with multidrug resistance strains. When these genes were knocked out, the strains were more antibiotic-sensitive, and the biofilm formation was significantly reduced [96].

### 6.4. The Roles of eDNA and Horizontal Gene Transfer

eDNA may also affect antibiotic resistance by altering the surrounding environment. The eDNA is an anionic macromolecule that can chelate cations such as magnesium ions, causing a decrease in the effective concentration of Mg^2+^ in the atmosphere [97]. eDNA may also physically interact with antimicrobials and try to impound the agent from the sequence targets. It also facilitates the horizontal gene transfer of resistance genes within the competent cells in the biofilm [97]. However, the direct mechanisms of eDNA on antibiotic resistance need to be considered in *H. pylori* for further understanding.

Horizontal gene transfer is another critical aspect of biofilm-associated antibiotic resistance, suggesting an evolutionary strategy to adapt to diverse environments. Vertical gene transfer of chromosomes or plasmids by cell division can be energy expensive; hence, the utility of horizontal gene transfer is more favorable. *H. pylori* have conjugation tools such as the Type IV secretion system and pilis [98,99]. This process involves the transfer of genetic material between bacterial cells, allowing for the exchange of antibiotic resistance genes and other beneficial traits. In biofilms, the structure includes vesicles that protect nucleic acids from degradation, enabling the sharing of antibiotic-resistant genes among bacteria [63]. A previous study reported that exposure to clarithromycin increased biofilm formation with a point mutation in locus 2142 or 2143 of 23S rRNA [66].

### 6.5. Lipid Modification

The human immune system generates antimicrobial compounds, such as calprotectin, to counteract infections. *H. pylori* can alter lipids to avoid being detected by the immune system and interact with calprotectin when cultured together, resulting in changes to enzymes such as LpxF, LpxL, and LpxR. These alterations lead to a higher amount of biofilm biomass, which assists in enhancing bacterial defense systems [100]. In addition, *H. pylori* can alter cholesterol to increase its ability to attach to stomach epithelial cells, contributing to its ability to cause disease [101]. The practical attachment to the epithelium is necessary for biofilm formation.

### 6.6. CRISPR-like Region

Previous studies explored the association between CRISPR-like regions and biofilm formation [91]. Among the isolates, 23 were CRISPR-like positive, with 19 capable of forming biofilms. However, contrary to previous studies, statistical analysis did not reveal a significant relationship between CRISPR-like regions and biofilm formation. The study findings diverged from the existing literature, highlighting the role of CRISPR systems in biofilm regulation in other bacterial species. For instance, CRISPR targeting the *luxS* gene inhibited biofilm formation in *E. coli*, while Cas genes in *P. aeruginosa* and *Salmonella* were implicated in biofilm production [21,102]. Additionally, a recent study reported that CRISPR deficiency in *S. agalactiae* led to decreased biofilm formation. These results shed light on the complex interplay between CRISPR-like regions and biofilm formation in *H. pylori*, providing valuable insights into the mechanisms underlying biofilm development in this pathogen.

## 7. Discussion: Alternative Solution Against the Biofilm and Resistance

The biofilm formation mechanisms mentioned above could be the potential target for biofilm prevention and *H. pylori* eradication. The biofilm has reportedly inhibited and decreased the function of commonly used antibiotics. The battle against *H. pylori* infections has spurred innovative research on novel treatment modalities. Therefore, exploration of some alternative compounds is required. For instance, extracts from *Rubus idaeus* have been noted for their bioactive compounds that may inhibit *H. pylori* proliferation, with studies indicating that polyphenols from these plants can exert cytotoxic effects on cancer cells, suggesting a broader therapeutic potential [103].

Additionally, probiotics such as *Lactobacillus plantarum* and *Lactobacillus salivarius* have effectively reduced *H. pylori* adhesion and inflammation by competitively excluding pathogens [104,105]. Moreover, compounds like curcumin, found in turmeric, and eugenol, derived from clove oil, have also been investigated for their ability to combat *H. pylori*. Curcumin exhibits anti-inflammatory properties, potentially weakening the biofilm structure and making it more susceptible to other treatments [106]. Another compound reported was Quinone Derivatives (Porphyromon and Phillygenin), which can generate reactive oxygen species (ROS). ROS can disrupt the biofilm matrix and lead to dispersal [107].

In addition to discovering new alternative drugs from natural resources, improving the specificity and effectiveness of the drug delivery system is also necessary. *H. pylori* colonizes a particular area in the gastric pit and is frequently washed with the acid. Therefore, a drug delivered and activated in the target can reduce unnecessary side effects to other healthy tissue. Nanotechnology has played a significant role, and researchers have explored various approaches. The most recent study utilized mucus-penetrating nanoplatforms as ultrasound-induced free radical initiators to specifically target *H. pylori* [108]. Another study showcased the efficacy of rubropunctatin-silver composite nanoliposomes and resveratrol-loaded chitosan nanoparticles in eradicating *H. pylori* both in vitro and in vivo [109,110]. Furthermore, exploring biofilm disruption using nanostructured lipid carriers and fullerenol nanoparticles has opened new avenues for eradicating *H. pylori* infections [111]. A study investigating nanoparticles targeting quorum sensing also showed a potential strategy to combat biofilms [112]. Moreover, developing gastric acid-responsive ROS nanogenerators and residence time-extended nanoparticles has offered innovative strategies for treating *H. pylori* infections without disrupting the intestinal flora [113,114].

The barrier of mucus formed by biofilms can also hinder drug deliveries, as discussed in the previous section. Developing multifunctional vesicles and copper-bearing metal–organic frameworks with mucus-penetrating capabilities has shown promise in effectively clearing mucosal colonized *H. pylori* [115]. Additionally, using blue light-emitting diodes and antimicrobial peptides has successfully targeted biofilm and multidrug-resistant strains of *H. pylori* [116]. In the quest for effective treatments, the synergy between natural compounds like Armeniaspirol A and synthetic agents has shown potential in combating drug-resistant strains of *H. pylori* [117]. Utilizing antimicrobial peptides, chitosan-albumin-based nano-antimicrobials, and electrolyzed superoxidized solutions has provided new avenues for addressing biofilm-related infections. The research landscape continues to evolve with studies focusing on the genetic determinants of biofilm formation, the role of bacterial cellulose chemisorbed with anti-metabolites, and the impact of phyto anti-biofilm elicitors on *H. pylori* [118]. To eradicate biofilms and *H. pylori*, Antimicrobial Photodynamic Therapy (aPDT) has been suggested as an alternate therapy approach for eliminating bacterial infections, notably effective against *H. pylori* due to its ability to manufacture and accumulate photosensitizing porphyrins [119]. These advancements underscore the diverse approaches explored to combat *H. pylori* infections, offering hope for more effective and targeted treatment options in the fight against this persistent pathogen.

## 8. Future Research

While numerous factors have been identified as contributing to *H. pylori* biofilm formation, the precise mechanisms and key targets for eradication remain unclear. Future research should integrate "the bench and the bedside “approach, integrating laboratory-based studies with clinical trials to bridge the gap between theoretical knowledge and practical applications. While numerous factors have been identified as contributing to *H. pylori* biofilm formation, the precise mechanisms and critical targets for eradication remain unclear. Advances in omics sequencing, bioinformatics, and machine learning can accelerate the identification of potential drug targets by analyzing the interactions between antibiotics or antibiofilm drugs and bacterial proteins. These predicted targets can then be validated through wet lab experiments.

Furthermore, the role of dispersal systems in biofilm removal and the involvement of outer membrane vesicles (OMVs) in mutation, horizontal gene transfer, and biofilm induction require further exploration. The efflux pump, which commonly occurs in biofilms and mainly contributes to antibiotic resistance, still has limited causal proof [120]. Further research is necessary to explore the potential of modifying the extracellular polymeric substance (EPS) matrix as a novel approach to disrupt established biofilms and enhance bacterial susceptibility to antibiotics [82].

In clinical settings, the unstable nature of *H. pylori* biofilms and their location within the gastric pit can pose challenges for visualization and studying their impact on patient outcomes. However, evaluating biofilm presence in patients with multidrug-resistant or highly resistant strains is crucial to establish a direct link between biofilms and clinical outcomes. A study involving clinical subjects could facilitate the investigation of multispecies biofilms from the gastric microbiome. Clinical trials for alternative drugs and delivery systems targeting *H. pylori* biofilms are essential for translating laboratory findings into effective treatments.

## 9. Conclusions

The biofilm is a significant cause of persisting *H. pylori* infection. Our current review provides an update regarding *the H. pylori* biofilm formation mechanism and roles in antibiotic resistance and explores an alternative solution for the drug delivery system. Further studies are required since *H. pylori* infection and gastric-cancer-related deaths remain high. Future studies should focus on the complex interaction within the biofilm, optimizing drug delivery, and exploring novel therapeutic agents. Addressing these challenges is essential to reducing the burden of *H. pylori*-related diseases and improving patient outcomes.

## Supplementary Materials

The following supporting information can be downloaded at: https://www.mdpi.com/article/10.3390/antibiotics13100976/s1, Table S1: Articles about Mechanism of Biofilm Formation in *Helicobacter pylori*.
