# Supplementary material for: Molecular Mechanisms of Biofilm Formation in Helicobacter pylori
Source: Antibiotics (Basel). 2024 Oct 16;13(10):976. doi: 10.3390/antibiotics13100976 (PMC11504965; doi:10.3390/antibiotics13100976)
Supplement: Supplementary file 1 [file antibiotics-13-00976-s001.zip › antibiotics-3210630-supplementary.pdf]

Supplementary Table S1. Articles about Mechanism of Biofilm Formation in *Helicobacter pylori*

Title	Potential Mechanism	Author	Year	DOI
CRISPR-like sequences association with antibiotic resistance and biofilm formation in Helicobacter pylori clinical isolates	CRISPR-like Sequences	Yousefi L, et Al	2024	10.1016/j.heliyon.2024.e26809
Counterclockwise rotation of the flagellum promotes biofilm initiation in Helicobacter pylori	Flagellar role in biofilm	Liu X, et al	2024	10.1128/mbio.00440-24
The Association of Helicobacter pylori Biofilm with Enterovirus 71 Prolongs Viral Viability and Survival	Biofilm increase the susceptibility of organism	Hassanbhai AM,et al	2023	10.3390/ijms241914500
Genetic determinants of Biofilm formation of Helicobacter pylori using whole-genome sequencing	Multiple genetic factors contributed to the biofilm formation	Fauzia KA, et al	2023	10.1186/s12866-023-02889-8
SpoT-mediated NapA upregulation promotes oxidative stress-induced Helicobacter pylori biofilm formation and confers multidrug resistance	SpoT mediated NapA induce biofilm	Zhao Y, et al	2023	10.1128/AAC.00152-21
Polymorphism of virulence genes and biofilm associated with in vitro induced resistance to clarithromycin in Helicobacter pylori	Virulence genes such as Cag4 contribute to biofilm formation	Rosli NA, et al	2023	10.1186/s13099-023-00579-4
Characterization of a Helicobacter pylori strain with high biofilm-forming ability	toxin antitoxin gene type II and multiple genes contribute to high biofilm strain	Wilkinson D, et al	2023	10.1099/jmm.0.001710

Comparative genomics analysis of statistically significant genomic islands of Helicobacter pylori strains for better understanding the disease prognosis	fliD operon was found to be connected to flagellar assembly and biofilm production.	Chakraborty J, Chatterjee R.	2022	10.1042/BSR20212084
Biophysical characterization of the homodimers of HomA and HomB, outer membrane proteins of Helicobacter pylori	HomA and HomB contribute to biofilm formation	Tamrakar A, et al	2021	10.1038/s41598-021-04039-4
Genetic requirements and transcriptomics of Helicobacter pylori biofilm formation on abiotic and biotic surfaces	Multiple genetic factors contributed to the biofilm formation	Hathroubi S, Hu S, Ottemann KM.	2020	10.1038/s41522-020-00167-3
Helicobacter pylori Biofilm Confers Antibiotic Tolerance in Part via A Protein-Dependent Mechanism	Extracellular protein play roles in biofilm and resistance	Hathroubi S, Zerebinski J, Clarke A, Ottemann KM.	2020	10.3390/antibiotics9060355
Identification and characterization of the α-CA in the outer membrane vesicles produced by Helicobacter pylori	α-CA in the outer membrane vesicles	Ronci M, et al	2019	10.1080/14756366.2018.1539716
Detection and Quantification of eDNA-Associated Bacterial Membrane Vesicles by Flow Cytometry	eDNA-Associated Bacterial Membrane Vesicles	Puca V, et al	2019	10.3390/ijms20215307
Agent-Based Modeling Demonstrates How Local Chemotactic Behavior Can Shape Biofilm Architecture	Chemotactic factor shape biofilm	Sweeney EG, Nishida A, Weston A, Bañuelos MS, Potter K, Conery J, Guillemin K.	2019	10.1128/mSphere.00285-19
A proposed role for diffusible signal factors in the biofilm formation and morphological transformation of Helicobacter pylori	Diffusible signal factors in the biofilm	Krzyżek P, Gościniak G.	2018	10.5152/tjg.2017.17349

Metabolomic analysis of low and high biofilm-forming Helicobacter pylori strains	Biofilm has low metabolism	Wong EHJ, Ng CG, Goh KL, Vadivelu J, Ho B, Loke MF.	2018	10.1038/s41598-018-19697-0
Morphology of Helicobacter pylori as a result of peptidoglycan and cytoskeleton rearrangements	Peptidoglycan and cytoskeleton role in biofilm	Krzyżek P, Gościniak G.	2018	10.5114/pg.2018.78284
Effect of Helicobacter pylori biofilm formation on susceptibility to amoxicillin, metronidazole and clarithromycin	Biofilm increase antibiotic resistant	Yonezawa H, Osaki T, Hojo F, Kamiya S.	2019	10.1016/j.micpath.2019.04.030
Helicobacter pylori Biofilm Formation Is Differentially Affected by Common Culture Conditions, and Proteins Play a Central Role in the Biofilm Matrix	Culture condition affect biofilm matrix	Windham IH, et al	2018	10.1128/AEM.00391-18
ArsRS-Dependent Regulation of homB Contributes to Helicobacter pylori Biofilm Formation	ArsRS-Dependent Regulation of homB	Servetas SL, et al	2018	10.3389/fmicb.2018.01497
Bifunctional Enzyme SpoT Is Involved in Biofilm Formation of Helicobacter pylori with Multidrug Resistance by Upregulating Efflux Pump Hp1174 (gluP)	SpOT and Efflux Pump Hp1174 (gluP) contribute to the resistance	Ge X, Cai Y, Chen Z, Gao S, Geng X, Li Y, Li Y, Jia J, Sun Y.	2018	10.1128/AAC.00957-18
Diversification of the AlpB Outer Membrane Protein of Helicobacter pylori Affects Biofilm Formation and Cellular Adhesion	AlpB Outer Membrane Protein	Yonezawa H, et al	2017	10.1128/JB.00729-16
Gene Expression Profiling of Transcription Factors of Helicobacter pylori under Different Environmental Conditions	Transcription Factors of *H. pylori*	De la Cruz MA, et al	2017	10.3389/fmicb.2017.00615

Molecular and Proteomic Analysis of Levofloxacin and Metronidazole Resistant Helicobacter pylori	Multiple genetic factors contributed to the biofilm formation	Hanafi A, et al	2016	10.3389/fmicb.2016.02015
Comparative Genomics Revealed Multiple Helicobacter pylori Genes Associated with Biofilm Formation In Vitro	Multiple genetic factors contributed to the biofilm formation	Wong EH, , et al	2016	10.1371/journal.pone.0166835
Detection of Pathogenic and Non-pathogenic Bacteria in Drinking Water and Associated Biofilms on the Crow Reservation, Montana, USA	Biofilm increase bacterial survival	Richards CL, et al	2018	10.1007/s00248-015-0595-6
Helicobacter pylori Biofilm Involves a Multigene Stress-Biased Response, Including a Structural Role for Flagella	Multigene Stress-Biased Response and Flagella	Hathroubi S, Zerebinski J, Ottemann KM.	2018	10.1128/mBio.01973-18
Influence of Helicobacter pylori culture supernatant on the ecological balance of a dual-species oral biofilm	Culture condition affect biofilm matrix	Zhang W, Deng X, Zhou X, Hao Y, Li Y.	2018	10.1590/1678-7757-2017-0113
Effect of biofilm formation by clinical isolates of Helicobacter pylori on the efflux-mediated resistance to commonly used antibiotics	Efflux pump play roles in biofilm and resistance	Attaran B, Falsafi T, Ghorbanmehr N.	2017	10.3748/wjg.v23.i7.1163
Identification of Factors Associated with Biofilm Formation Ability in the Clinical Isolates of Helicobacter pylori	Multiple genetic factors contributed to the biofilm formation	Attaran B, Falsafi T.	2017	10.15171/ijb.1368
Biofilm formation enhances Helicobacter pylori survivability in vegetables	Biofilm increase bacterial survival	Ng CG, Loke MF, Goh KL, Vadivelu J, Ho B.	2017	10.1016/j.fm.2016.10.010
